# Vehicles, Replicators, and Intercellular Movement of Genetic Information: Evolutionary Dissection of a Bacterial Cell

**DOI:** 10.1155/2012/874153

**Published:** 2012-04-10

**Authors:** Matti Jalasvuori

**Affiliations:** ^1^Department of Biological and Environmental Science, Center of Excellence in Biological Interactions, University of Jyväskylä, 40014 Jyväskylä, Finland; ^2^Division of Evolution, Ecology and Genetics, Research School of Biology, Australian National University, Canberra, ACT 0200, Australia

## Abstract

Prokaryotic biosphere is vastly diverse in many respects. Any given bacterial cell may harbor in different combinations viruses, plasmids, transposons, and other genetic elements along with their chromosome(s). These agents interact in complex environments in various ways causing multitude of phenotypic effects on their hosting cells. In this discussion I perform a dissection for a bacterial cell in order to simplify the diversity into components that may help approach the ocean of details in evolving microbial worlds. The cell itself is separated from all the genetic replicators that use the cell vehicle for preservation and propagation. I introduce a classification that groups different replicators according to their horizontal movement potential between cells and according to their effects on the fitness of their present host cells. The classification is used to discuss and improve the means by which we approach general evolutionary tendencies in microbial communities. Moreover, the classification is utilized as a tool to help formulating evolutionary hypotheses and to discuss emerging bacterial pathogens as well as to promote understanding on the average phenotypes of different replicators in general. It is also discussed that any given biosphere comprising prokaryotic cell vehicles and genetic replicators may naturally evolve to have horizontally moving replicators of various types.

## 1. Introduction

Viruses that infect prokaryotic cells are known to be enormously diverse in terms of genetic information [1, 2]. Most novel viral isolates are likely to have at least some genes that have no homologues among any of the previously known genes, including those in the genomes of related viruses [3]. Yet, there has been a dispute whether or not new genes may actually emerge in viruses [3]. Viruses are dependent on cellular resources such as nucleotides, amino acids, and lipids for producing more viruses; therefore it seems justified to ask whether they also use cellular genes for their genetic information. Yet, when viral genes are compared to other genes in databases, it often appears that they have no cellular counterparts [2]. Where then do these viral genes come from? Have they been acquired from a cellular host that we simply have not sequenced before? Or alternatively, are the cellular genes perhaps just evolving rapidly in viral genomes so that their common ancestry with the host genes can no longer be derived? Or perhaps, is it indeed possible that new genes actually emerge in viruses themselves?

Forterre and Prangishvili from Pasteur Institute argued that the core of the dispute appears to be in the notion that viruses are often considered to be just their protein-encapsulated extracellular forms [4] that are only stealing cellular resources (including genes) for their own purposes [3, 5, 6]. Take any textbook on viruses and majority of the pictures representing viruses are of the various types of viral shells composed of proteins (and sometimes lipids) that enclose the viral genome. But these infectious virus particles, or virions, are inert in all respects unless they encounter a susceptible host cell [7]. And due to this inertness of virions it is difficult to understand how a virus could ever come up with completely new genes. 

The answer is, naturally, that viruses cannot produce new genes during their extracellular state, and thus any potential event for the emergence of a new viral gene must still occur within a cell during the replication cycle of a virus [5]. But if the gene emerges in the genome of a virus, then would it rather be the virus, and not the cell, that was the originator of that gene? Or, to put it differently, was it not the virus that benefited from the emergence of new genetic information? The actual process that causes the genetic information to acquire the status of a gene would still be due to similar processes as the origin of genes within chromosomes (these being different types of genetic changes, such as point mutations, insertions, deletions, gene duplications, etc.), but these changes would be selected due to their improvements on the fitness of the virus. This reasoning has made Forterre to propose a model where viruses are seen essentially as a cellular life form that can also have an extracellular state [7, 8]. Virus is not strictly equivalent to the protein-enclosed viral genome. Rather, the extracellular form of a virus should be denoted as a virion, and this virion should not be mistaken for a virus. Viruses, in a complete sense, are organisms that live within cells (i.e., ribosome-encoding organisms) and can transform other cells into virus-cell organisms by producing more virions. In other words, viruses can utilize an extracellular encapsulated form to transfer its genetic information from one cell to another. Forterre coined a term *virocell*, which refers to the stage of viral life during which the virus is within a cell [7]. The virocell organism is indeed both a (capsid encoding) virus and a (ribosome encoding) chromosome, and the actual phenotype of the virocell is encoded by both of these genetic entities. The virocells are entirely capable of coming up with novel genetic information just as cells are, and thus approaching viruses from this perspective should clear any controversies about the emergence of new genetic information in viruses.

Forterre's line of reasoning along with my own studies on various different genetic elements (including characterization of temperate and virulent viruses [9, 10]; determination of common ancestor between plasmids, viruses and chromosomal elements [11]; conduction of evolution experiments with bacteria, viruses, and plasmids [12, 13]; as well as more theoretical work on horizontal movement of genetic information [14, 15]) has served as an inspiration for this paper. Indeed, it could be argued in more general terms what it means that prokaryotic cells can be (and often are) chimeras of various types of genetically reproducing elements. Virocell concept clears effectively many of the confusions between viruses and virions and their relationship with cells. Nonetheless, virocell is only a special case among all the possible types of prokaryotic organisms. Bacterial and archaeal cells can also contain conjugative plasmids, various types of transposons, defective prophages, and many other independent replicators that are distinct from the ribosome encoding prokaryotic chromosome. Together these replicators can produce organisms in all possible combinations. In order for the arguments about virocells to be consistent with the other potential chimeras of genetic replicators, the cell itself must be considered as a separate entity from all the genetic replicators (including chromosomes) that exploit the cell structure for replication. In the following chapters I will perform an evolutionary dissection to a bacterial cell. This will lead into the separation of cell vehicles and replicators from each other and thus provide one potential way to approach the evolution of bacterial organisms.

## 2. Vehicles and Replicators

“*A vehicle is any unit, discrete enough to seem worth naming, which houses a collection of replicators and which works as a unit for the preservation and propagation of those replicators*”, Richard Dawkins wrote in *Extended Phenotype*. Dawkins utilized the concepts of replicators and vehicles in an argument which stated that evolution ultimately operated on the level of genetic information and not on the level of populations of organisms, species, or even cells. Replicators refer to packages of genetic information that are responsible for any effective phenotype of the vehicle. Vehicle itself can be a cell, a multicellular organism, or, for example, the host organism of a parasite. “*A vehicle is not a replicator*”, argued Dawkins in an attempt to underline that it is the replicator (like the chromosome of a parasite) and not the vehicle (like the parasitized cell) that evolves. This difference, however, may sometimes be seemingly trivial, which is why it has caused some dissonance among evolutionary biologists.

Nevertheless, Dawkins' work focused mostly on explaining evolutionary issues of eukaryotic organisms, but the replicator-centered evolution naturally operates also within and between prokaryotic cells. Indeed, there is a vast diversity of different forms of genetic replicators that use prokaryotic cell vehicles for their preservation and propagation. Any particular prokaryote that lives in this biosphere, being that a bacterium on your forehead or an archaeon in the bottom of Pacific Ocean, harbors a chromosome but may also host a collection of other replicators, including plasmids, transposons, and viruses. Some of the replicators, like conjugative plasmids and viruses, are able to actively move between available vehicles in its environment, thus making these replicators less dependent on the survival of any particular lineage of cell vehicles. Therefore they are not an inherent part of any particular bacterium and may thus be considered as distinct forms of genetically replicating entities that utilize cells for their propagation and survival (similarly with the viruses in Forterre's virocell concept).

The continuous struggle for existence within and between prokaryotic vehicles modifies the phenotypes of the replicators. A lot of theoretical and experimental work has been done in order to clarify the functions and the evolutionary trajectories of viruses, bacterial cells, and plasmids in different ecological contexts and under various selection pressures. However, in this discussion I take a step away from any particular type of a replicator or an organism and explore from a general perspective whether the lateral movement potential (or lack of it) of the replicators could help illuminate some evolutionary aspects of the prokaryotic biosphere. This discussion attempts to provide an intuitive view on the selfish genes and various types of replicators in bacterial and archaeal cells. It is my intention to keep the text simple and readable regardless of the reader's expertise on bacteria, viruses, plasmids, or, for that matter, evolutionary theory. Moreover, given the vast amount of details in microbial world, I hope that the readers realize that certain corners had to be cut in various places in order to keep the text within realistic length.

**Figure 1:**
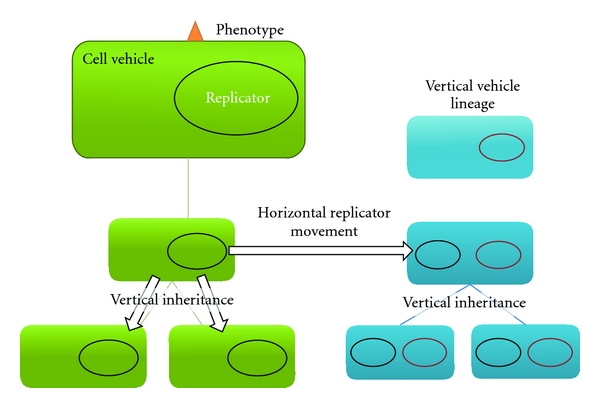
The basic terminology used throughout the paper and their biological counterparts.

Furthermore, in an attempt to maintain the simplicity, the following nomenclature and definitions are used throughout this paper. A *cell vehicle* denotes a prokaryotic cell with membranes, resources, and everything else but excludes any genetic material. *Cell-vehicle lineage* indicates a single vehicle and its direct descendant that emerge by cell division. A *replicator *is any discrete enough collection of genetic material (that seems worth naming), which utilizes the cell vehicle for its preservation and propagation. Replicators are replicated as distinct units forming a coherent collection of genetic material that can be separated with reasonable effort from other replicators. Replicators may be replicated as a part of the replication of other replicators, as integrative viruses are replicated along with host-chromosome multiplication, but essentially these two replicators can be denoted as two distinct entities given that the integrative virus can replicate its genetic information also separately from the replication of the chromosome. The mean by which the genetic information of a replicator is replicated is not relevant. However, I prefer to not make a too strict definition for a replicator as it is likely to lead to unproductive hair-splitting arguments. Yet, it must be noted that replicators do not include ribosomes or other nucleic acids containing molecules that essentially have an enzymatic function but that are not used as template for their own replication*. Vertical relationship* or vertical inheritance of a replicator indicates that this genetic replicator preserves itself within a dividing lineage of cell vehicles. *Horizontal movement potential* denotes that the replicator is able to introduce itself into a cell-vehicle lineage where the replicator was previously absent. Any feature that is encoded or induced by a replicator is denoted as a *phenotype*.  Figure 1 links these terms with their biological counterparts.

## 3. Laterally Moving Replicators

Prokaryotic world contains a number of different types of replicators that have potential for lateral movement between cell-vehicle lineages. Here I briefly introduce the basic types of laterally moving replicators.

### 3.1. Conjugative Plasmids

Conjugative plasmids are extrachromosomal assemblies of genetic material that replicate independently within their host vehicles [16, 17]. Conjugative plasmids may encode complex toxin-antitoxin systems and other effectors that ensure that the dividing cell vehicles harbor copies of the plasmid [18]. Conjugative plasmids also encode proteins that facilitate the transfer of the conjugative plasmid from one cell vehicle to another [19]. Conjugative plasmids can spread between distantly related cell vehicles, but one copy of the plasmid is always maintained within the donating cell. Conjugative plasmids have no extracellular stage and are thus dependent on the host cell at all times.

### 3.2. Integrative and Conjugative Elements (ICEs)

Similarly with conjugative plasmids, ICEs can force the host cell vehicle to form a cell-to-cell contact with other cells in the present environment and use this contact for transporting the genetic element from one cell to another [16, 20, 21]. ICEs can spread between distantly related cell vehicles and replicate therein. ICEs integrate into the chromosome during their life cycle and differ from conjugative plasmids in this respect. This integration may often lead to the transfer of some chromosomal genes from one host to another.

Conjugative plasmids and ICEs are known for their antibiotic resistance genes [22]. Arguably the lateral movement of conjugative plasmids and ICEs is responsible for majority of novel drug-resistant bacterial phenotypes in hospitals and other clinically important environments [16]. Conjugative plasmids and ICEs contain variety of different types of genes including those encoding for virulence factors. However, detailed analysis of this genetic variability and their exact functions and/or roles in certain ecological contexts are beyond the scope of this paper.

### 3.3. Temperate Viruses

Viruses are replicators that enclose their genetic material within a protective protein capsid [3]. This capsid can leave the host cell and introduce the genetic material into a new cell vehicle far away from the initial host. Thus viruses (unlike plasmids) can be transiently independent from the survival of any particular host cell vehicle. The extracellular state of viruses is known as virion, and it should not be mistaken for a virus [7, 8]. Differences between virions and viruses were discussed in the introduction.

The assembly of viral particles often leads to the destruction of the cell vehicle. However, temperate viruses are able to exist peacefully within their host cell as a so-called provirus [23]. During the provirus state no viral particles are produced. Yet, this lysogenic cycle can be interrupted, which then leads into reigniting the virus particle production. Viruses can become integrated into the host chromosome or exist as extrachromosomal genetic elements during the provirus state [24–26]. A lysogenized cell vehicle is (usually) resistant to infections of other related viruses.

### 3.4. Virulent Viruses

Virulent viruses are incapable of lysogenic life cycle as they do not maintain regulation machinery that would allow them to retain from virus particle production. Virulent viruses destroy the infected cell vehicle at the end of their replication cycle. However, some virulent viruses can sometimes halt their replication cycle when the host cell is going into dormant state [27].

### 3.5. Passive Movement of Other Replicators

Prokaryotic cell vehicles can harbor other replicators that can occasionally move horizontally between cell-vehicle lineages, but they do not actively encode functions that would facilitate horizontal movement. These replicators include genetic elements like nonconjugative plasmids, and transposons. Plasmids, transposons, and even complete chromosomes can become transferred from one vehicle to another through the same conjugation channels that conjugative plasmids and ICEs use. Some plasmids or plasmid-like elements can spread from one cell to another within virus capsid [28] or cell-to-cell connecting nanotubes [29, 30]. Moreover, the natural competence of certain cell vehicles allows the uptake of foreign genetic material from the environment [31, 32], which can lead into horizontal movement of replicators between unrelated vehicle lineages. I will not perform thorough analysis of these various types of ways by which genetic information may become transferred between cell vehicles, but it is important to note that such events occur in natural systems.

## 4. Replicator Dependency on Vertical Survival of Cell Vehicles

Vertical lineage of a cell vehicle indicates a single prokaryotic cell vehicle and all of its direct descendants that emerge via cell division. If a replicator is exclusively dependent on the survival of a certain cell-vehicle lineage, the replicator would inevitably die along with the lineage. Chromosomes exemplify such a replicator. Yet, certain genes of chromosomes may become horizontally transferred even if the cell-vehicle lineage in general would go extinct (e.g., by transposon-induced transfer and recombination). However, for the clarity of this paper, all such (relatively) random potentials that are not general (enough) features of the replicators are being ignored.

Virulent viruses represent a class of replicators that are not bounded by the vertical survival of the cell vehicle. They even cause the demise of the particular cell vehicle as a part of their replication cycle. Yet, we must realize the limits of such definitions as these are just depictions of the average behaviors of biological entities within reasonable time frames. Naturally even virulent viruses are dependent on the survival of the particular vehicle they are infecting until new virus particles are completely assembled. They are also dependent on the existence of susceptible vehicles in the environment. Nevertheless, it can be argued that, due to their survival strategy, virulent viruses are not dependent on any particular vehicle.

All other replicator types, like plasmids and temperate viruses, are intermediates between virulent viruses and chromosomes in respect to their dependencies on the vertical survival of their current vehicles. This relationship between replicators and vehicles is, naturally, reciprocal as the cell vehicle is not able to survive in absence of the chromosome whereas it fails to survive in presence of a virulent virus. Interestingly, however, this seemingly trivial notion allows us to position the different replicators on a scale where their dependency on cell vehicle appears to (negatively) correlate with their vehicle survival affecting phenotypes (see Section 10). In other words, it is possible that the average phenotype of any replicator matches its position on a chart where lateral movement is on one axis and the vehicle-benefitting phenotype is on the other. Of course, this is just a rough approximation and only an artificial depiction of the result of natural selection repeatedly acting on the replicators. Yet, it can provide a tool to describe the average behavior of prokaryotic replicators. Before addressing this aspect in greater detail, we need to analyze and classify the replicators in a more definitive manner.

## 5. Classification of Replicators

Most (if not all) of the different types of replicators that utilize prokaryotic cell vehicles for preservation and propagation can be classified according to their horizontal movement potential between individual cell-vehicle lineages and according to their vertical dependencies on cell vehicles. I will attempt to argue that certain phenotypic traits usually associate with replicators of the same class. Subsequently I will discuss the reasons behind this by analyzing few hypothetical scenarios where natural selection might favor the association of these phenotypic traits with horizontally moving replicators rather than with strictly vertically evolving chromosomes. The classification is presented in Table 1.

My attempt was to retain the classification as simple as possible while maintaining the essential insights that may be derivable from it. Yet, it must be noted that strict boundaries cannot be drawn between different classes because this classification seeks to group together highly different and usually unrelated biological entities. Indeed, there are numerous cases where replicators have changed their present classes and have done this rapidly in evolutionary terms. For example, many chromosome-integrating proviruses (*Class IV*) are known to have become defective viruses by conjoining with a* Class I* replicator (chromosome) and thus becoming only a vertically inherited element [23]. Conjugative plasmids (*Class III*) are known to have become conjugation-defective plasmids (turning into *Class II* replicators) [13], and it has been noted that homologous genetic elements can belong to multiple different classes [10, 11]. In other words, the classes do not represent any permanent characteristics of the replicators. Therefore it seems appropriate to ask whether assignment of replicators into any of the classes is able to catch any practical attributes of an evolving biosphere (and thus justify its formulation). In the remainder of the paper I will attempt to address this question from few different perspectives. For example, I will argue (with some examples) that by changing a *class* the replicator starts to evolve towards other replicators within that group. This suggests that repeated rounds of selection on the replicator can have a general trend in shaping the replicator into a typical member of its *class*. Nevertheless, the complexity of the actual natural systems and the shortcomings of such classifications in relation to this complexity are discussed to some extent.

Finally, I want to emphasize that this classification only attempts to provide a tool to improve our means for understanding and discussing the evolution of prokaryotes and their genetic elements. Some might find the classification trivial or obvious, but I believe that it can help some of us simplify the vastly diverse prokaryotic world into evolutionarily useful components. In any case, there are various types of genetic elements in this biosphere that express different phenotypes and vary in their potential for horizontal movement between cells. It seems very likely that the phenotype is associated at least for some parts with the movement potential. Assuming the opposite (that the horizontal movement potential is not related with the phenotype) seems impossible as you may consider the vehicle-terminating virulent viruses as an example of an expressed phenotype (the only mean by which a virulent virus may survive is due to its horizontal movement between vehicles). Therefore, whether we find it practical or not, it is possible to group these features to some extent (regardless of the usefulness of the presented classification). Naturally some extensions to the presented classification (like, e.g., inclusion of the notion of plasmid incompatibility with each other and with some chromosomes [33]) can be introduced, if found necessary. However, I tried to avoid any unnecessary complexity in order to keep the classification intuitively comprehensive.

## 6. Phenotypic Traits of Replicators

In this section I will go through the usual phenotypic traits of each replicator *classes*. However, it must be noted there are many replicators that have minor or major exceptions to the general traits within each *class*. In other words, replicators in general form a highly diverse group of genetic entities that utilize cell vehicles for replication and preservation in various environments and in various ecological contexts. Yet, general approximations may be done to some extent.

### 6.1. *Class I*: Prokaryotic Chromosomes

Chromosomes are the main genetic replicator in cell vehicles. It segregates into both daughter cells during division. It is often considered that any prokaryotic cell is “equal” to its chromosome. Indeed, when studies attempt to identify the genus of a bacterium, the ribosomal genes or some other highly essential chromosomal genes are selected for sequencing. By determining the divergence of sequence of that gene in comparison to other homologous genes in other cell vehicles, it is possible to assign the taxonomic position of the bacterium. This indicates that many chromosomal genes are absolutely essential for the survival of the cell vehicle, and therefore they can be reliably used to determine the evolutionary histories of both the chromosomes and their corresponding cell vehicles (even if I here treat chromosomes and vehicles as distinct and separate components of a cell organism).

The survival of the chromosome replicator is tightly interlocked with the survival of its current cell vehicle. Natural selection favors any phenotypic change in the chromosome that improves the reproductive success and survival of the cell vehicle. In other words, a favorable mutation (or other genetic change) in a chromosome should not decrease the fitness (or increase the reproductive cost) of the cell vehicle. However, evolutionary process within actual populations of prokaryotes is very complex process (even if other replicator types are not involved), and selection may operate on levels above individual cell vehicles. Yet, for the purposes of this discussion, the correlation of the fitness of the chromosomal replicator with the fitness of the cell vehicle is satisfying enough.

### 6.2. *Class II*: Plasmids and Transposons

Plasmids are circular or linear DNA molecules that replicate independently to chromosomes within cell vehicles. However, plasmids always require certain genetic products of chromosomes (being those ribosomes, DNA polymerases, or something else). The sizes of their genome vary from a few kilobases to hundreds of thousands of bases.

Plasmids rely on few different strategies to ensure their survival within the dividing host vehicles. They can encode molecular mechanisms that separate the plasmids along with the chromosomes. Some plasmids contain genes for a toxin-antitoxin system. Plasmid encodes both a stable toxin and unstable antitoxin. The stable toxin will destroy host vehicle, if the vehicle does not contain a copy of the antitoxin-producing plasmid. The plasmids that have either segregation or toxin-antitoxin system (or both) usually control the copy number of plasmids within cell vehicles [34]. These plasmids are large in their size, and thus each copy of the plasmid is a burden to the general reproductive rate of the cell vehicle. Similarly with temperate viruses, plasmids are able to prevent other vehicles harboring similar plasmids to conjugate with their present vehicle [35]. 

Smaller plasmids may not encode sophisticated segregation mechanisms, but instead they can exist in high numbers within cell vehicles (tens to hundreds of copies) and are stably maintained due to the high probability that the dividing cell will contain a copy of the plasmid in both daughter cells.

Several studies have shown that the presence of plasmids in cell vehicles increases the reproductive cost of the cell. In other words, when cells without and with plasmids are grown in similar conditions, cells without plasmids are able to reproduce more rapidly. Moreover, cell vehicles themselves are generally not dependent on their plasmids. From this perspective it is obvious that the plasmid has to ensure its survival within the vehicle. Should the plasmids decrease the cost of reproduction of the cell vehicle, then selection would favor plasmid-containing cells over plasmid-free cells even without any encoded survival mechanisms. 

However and despite the general burden of plasmid, they can sometimes greatly improve the reproductive success of the cell vehicle. Antibiotic resistance genes are often part of plasmid replicators [16, 36]. Other plasmids have genes that help the cell vehicle utilize rare resources when nutrients are scarce. Plasmids can also encode toxins that help the cell vehicle destroy surrounding cells, like human tissues, and thus utilize the resources from these cells for their own benefit [37]. The reasons behind the existence of these genes in *Class II* (and *III*) replicators are discussed later.

### 6.3. *Class III*: Conjugative Plasmids and Other Conjugative Elements

Conjugative plasmids are extrachromosomal genetic elements similar to *Class II* plasmids. However, their existence within a cell vehicle changes the vehicle phenotype by such that the cell can form conjugation channel between its current vehicle and another vehicle in the surrounding environment. Through this channel *Class III *plasmid transfers itself into another cell vehicle. Conjugations put a reproductive cost on the hosting cell vehicle, and thus plasmids can regulate its repression as well as inhibit super-conjugation with vehicles that already contain a copy of *Class III* plasmid [35]. Conjugative elements can respond to the stress of the host vehicle, like the presence of antibiotics in the environment, and ignite transfer of the element to other vehicles [38].

Conjugative plasmids use similar and homologous mechanisms for their stable maintenance within vertical cell-vehicle lineages with nonconjugative (*Class II*) counterparts. *Class III* replicators often contain antibiotic resistance genes, and studies suggest that *Class III* replicators are the main cause behind the emergence of clinically relevant bacteria resistant to antibiotics [16].

### 6.4. *Class IV*: Temperate Viruses

Temperate viruses can produce virions, that is, the infectious virus particles, and therefore exist in a “dormant” state in the extracellular environment. However, they can also vertically coexist within cell-vehicle lineages along with *Class I*, *II,* and *III* replicators. Temperate viruses may integrate into the host chromosome and replicate as a part of *Class I* replicator during cell division. This integration, however, does not abolish the ability to move horizontally between vehicle lineages.

The genomes of temperate viruses may contain genes that are beneficial to the reproduction of their host vehicles (under certain conditions). Presence of a provirus can transform an avirulent bacterium into a virulent one by providing genes for different types of toxins [39]. These toxins can, for example, allow the bacterium to destroy host tissues. Proviruses may also change the host-vehicle phenotype so that it cannot be recognized by eukaryotic immune systems [40].

*Class IV* viruses are able to detect the malfunction, damage or stress of their host cell vehicles. Proviruses react to these signals by igniting the production of virus particles [41]. In other words, temperate viruses can predict the upcoming interruption of the vertical cell-vehicle lineage and readily progress into expressing their horizontally moving phenotype. As temperate phages are not dependent on the survival of the host vehicle, they often destroy the doomed vehicle themselves as a part of their lytic life cycle.

### 6.5. *Class V*: Virulent Viruses

Virulent viruses also produce virions and thus spend part of their life cycle in the extracellular environment as inert particles. Virulent viruses exclusively destroy the host vehicle as part of their life cycle. Virulent viruses generally do not contain genes that would benefit the vertical survival of the host cell-vehicle. The genetic content of *Class V* replicators appears to aim to effectively utilize the resources of cell vehicles in order to produce multiple horizontally moving virus particles. This, however, does not mean that virulent viruses are simple. Many lytic viruses, like T4, can independently encode essential functions such as some transfer RNA genes, and, indeed, T4 is one of the most complex bacteriophages described to date [42].

## 7. How Replicators Benefit from the Horizontal Movement between Vehicle Lineages?

Why should a replicator change or move to another vehicle lineage? It is not always obvious why the horizontal movement can be beneficial for a replicator. Indeed, without acknowledging the horizontal movement potential, it appears difficult to understand why bacterial cells or independent replicators have certain types of genes or phenotypes. By realizing that bacterial cells themselves are not always the actual units that are targeted by natural selection can help adopting a truthful image of the microbial world. In this section I consider few simple hypothetical cases that exemplify the effects of horizontal movement on the evolution of replicators and on bacterial organisms.

However, it must be pointed out that this section does not aim to provide any general models or prove any concepts, but instead it is an attempt to intuitively promote the way by which we see the replicators as dynamic components of cell-vehicle populations. The following scenarios are artificial, but their simplicity may help grasping the essence behind the evolution of horizontal movement potential.

### 7.1. Benefit of Being a Plasmid (*Class II* and *Class III*)

Imagine a world consisting of hundred independent cell-vehicle lineages. Each of these lineages contains only a single cell that reproduces as fast as it dies, keeping the number of each cell-vehicle type effectively at one. All the lineages replicate and die at identical rates in ultimate resources, and thus the proportions of each cell-vehicle type remains the same. In practice, there is no evolution in this system. By definition this means that the genetic composition of the population is not changing in respect to time.

However, assuming that one of the hundred lineages contains its genetic information in two independent replicators: a chromosome and a reproductively costless plasmid, given that the plasmid has a potential for horizontal movement between vehicle lineages, then the separation of these replicators into two distinct entities already brings evolution to the system.

**Figure 2:**
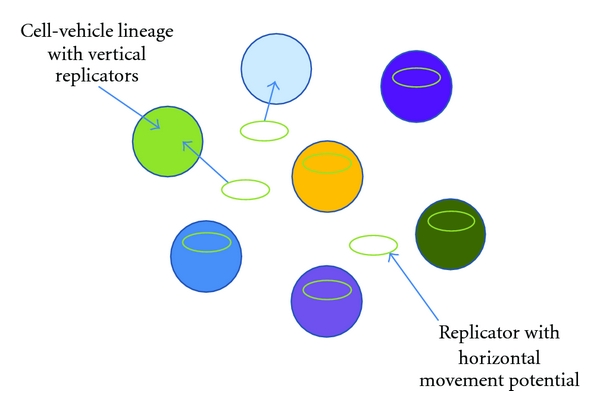
Replicators with horizontal movement potential can become common in various cell-vehicle lineages and therefore free of the survival of any particular lineage.

In the beginning the plasmid is present only in one percent of the cells in the world. Yet, sometimes after cell death the plasmid is released into the environment. From the environment it has a tiny chance to become introduced into a new cell-vehicle lineage. Each new transformed lineage increases the proportion of the plasmid by one percent and further contributes to the plasmid spread rate. Eventually the plasmid would be present in all of the cell vehicles, and therefore, in comparison to the initial chromosomal partner of the plasmid, the plasmid will be hundred times more successful in terms of prevalence among vehicle lineages. The simple existence of a replicator in an extrachromosomal form with tiny chance for horizontal movement has given it the potential to become by far the most abundant replicator in the system. This simple mind exercise can provide us with a glimpse of the underlying forces of natural selection that operates in actual biological systems. But why should natural selection favor the maintenance of the extrachromosomal form of the plasmid? Why not integrate with the chromosome after entering the cell? If some of the plasmids had permanently integrated to the chromosomes, they would have ceased transforming new cell vehicles into plasmid-containing lineages after the death of the bacterial organism. Thus, as long as there are plasmid-free vehicles available in the system, some of the plasmids may retain their extrachromosomal status as it facilitates the spread (as depicted in Figure 2).

Now consider how the introduction of reproductive cost on the plasmid replication would change the system. Or what if the plasmid somehow evolved a more effectively spreading phenotype and sometimes the plasmid could be lost due to segregation infidelity? Or if the plasmid contained genes that can sometimes increase the reproduction rate of the hosting cell vehicle while they put a general fitness cost on the host? Some of these questions are discussed below. Yet, such complexity is the reality of the ecological dynamics of plasmids in natural environments, and thus these mind games can only provide a platform from which to dive into the real world.

Nevertheless, it appears reasonable to assume that under certain conditions evolution may favor extrachromosomal genetic elements, such as plasmids, that can occasionally join previously plasmid-free cell vehicles. Plasmids also benefit from being as little reproductive cost to their host cells as possible. However, we immediately notice that the faster the plasmid can spread among plasmid-free cells, the faster it takes over the cell-vehicle populations. If there were hundred million cell-vehicle lineages instead of a hundred, even tiniest changes in the rate of spread would hugely affect the reproductive success of the plasmid (given some restricted time window for observing the success). Many studies have tackled the details of the interplay between the spread rates and reproductive costs of plasmids [19, 35, 43]. Theoretical work suggests that certain parameter values generally allow the stable maintenance of plasmids in a (sub) population of cell vehicles [44, 45]. Nevertheless, the rapid spread leads us to conjugative plasmids, which can actively force their host cell-vehicles to conjunct with plasmid-free cell vehicles in an attempt to transfer the plasmid.

Conjugative plasmids (generally) spread faster than nonconjugative plasmids, and thus, if the two plasmid types were equal in other respects, conjugative plasmids would apparently be evolutionarily more favorable plasmid type. However, the formation of conjugation channels between cell vehicles does not come without a reproductive cost. Indeed, evolutionary research of bacteria often focuses on studying such tradeoffs where one phenotype (e.g., conjugative) is favorable in certain conditions and the other phenotype (e.g., nonconjugative) in alternative conditions. In principle, the conjugative phenotype is practically useless if all cells in the population already harbor a copy of the conjugative plasmid and similarly highly useful when there are plenty of plasmid-free cell vehicles around [43]. Conjugative plasmids always require a cell-to-cell contact for plasmid transfer, indicating that only one (or few) cell(s) at the time can receive the plasmid.

However, as a mind exercise, consider a high copy number nonconjugative plasmid, which can release several plasmid replicators to the environment upon the death of the host vehicle. In principle, each of these replicators has a potential to become introduced into a new cell vehicle, and under ideal conditions high copy number plasmids could spread very fast in a plasmid-free population of cell vehicles. Yet, the naked DNA molecule is fragile in an extracellular environment and the uptake of the molecule requires a competence for plasmid intake from the cell vehicle. In other words, the plasmid will not survive long in the environment and it cannot force the cells to internalize the DNA molecule. Therefore it must be favorable from the perspective of the chromosome or other in-vehicle replicator (as they would encode the competent phenotype of the cell-vehicle) to introduce the new DNA molecule into the cell vehicle. Genes for antibiotic resistances and other beneficial functions can, under certain conditions, significantly increase the fitness of any cell-vehicle lineages. For this reason, opportunistic genes do not need to only improve the survival of their present vehicles but may sometimes also indirectly improve the probability by which the plasmid can spread horizontally to a new cell vehicle lineage and survive within that lineage thereafter. Natural competence, or the uptake of genetic material into the cell-vehicle from its vicinity, is as the name indicates a natural trait of many bacteria [46]. However, there are also many reasons why natural competence can backfire, and, supposedly, for this reason it is not prevalent trait among bacteria.

Nevertheless, plasmids may evolve mechanisms that allow them to hitchhike through conjugation channels build by other plasmids. This allows them to utilize the horizontal transfer potential without the burden of maintaining genetic machinery for it. Plasmids may also favor evolution towards higher copy numbers within a single cell vehicle in order for the highest copy-number plasmid to have the highest chance for getting transferred into new host vehicle. Yet, the increased cost of maintaining most copies can become compensated on population level by the lower reproductive cost that the lower copy-number plasmid put on individual vehicles [47]. As these different aspects hopefully demonstrate, the actual evolution of the phenotypes of plasmids is a complex subject in which several aspects must be considered. It is not immediately obvious which traits are favorable, and thus I want to retain here the more distant perspective on plasmids and other genetic elements.

### 7.2. Benefit of Being a Virus (*Class IV *and *V* Replicators)

In previous section it was considered how the release of high-copy-number plasmids into the environment could provide these replicators a high spread rate among vehicles, if the vehicles in the same environment are willing to take in these replicators. However, viruses are able to overcome this barrier of willing uptake by having the extracellular phenotype that forces the intrusion of the replicator into a suitable host vehicle.

Viral life strategy is dependent on the existence of suitable vehicles in the environment. However, given a susceptible population of cell vehicles, viral strategy is the fastest way by which the replicator can spread in the population. For this reason, all cellular organisms are under constant pressure to avoid viral infections. This, in turn, has led to the everlasting evolutionary arms race between viruses and their hosts [48, 49]. Viruses can obviously effectively maintain their life strategy despite the cost that they put on their current host. However, the ubiquity of viruses cannot be understood without taking the cell vehicles and the vehicle phenotypes into account. Indeed, virions, the extracellular forms of viruses, are the most abundant biological entities on our planet [6]. Yet, as Forterre has argued, virions themselves cannot be considered as living organisms in the same respect as cells can. Ultimately, viruses survive because their hosts survive [50].

### 7.3. Benefit of Being a Chromosome (*Class I* Replicator)

The existence of chromosomes in any cellular organism is so profound to our concept of cells that we might not even come to think of them as one of the replicators that utilize the cell vehicle for its propagation and preservation. However, in order to distinct the vehicles and replicators from each other under natural selection, we must also address the benefit (and cost) of being a strictly vertically inherited replicator (e.g., a chromosome). To emphasize the reality behind the distinction of replicators from vehicles, it was recently shown that the genome of one bacterial cell vehicle can be replaced by a (closely related) chromosome from another cell vehicle or by an artificially synthesized chromosome [51, 52]. This indicates that the concept of bacterial cell vehicles and their chromosomes is compatible with experiments and therefore their separation is not just a theoretical notion. I discuss here one possible way to approach the evolution of replicators towards a strictly vertical phenotype.

As stated before, all replicators are dependent on cell vehicles for their propagation. The actual living systems have limited resources, and thus the number of cell vehicles rapidly advances to its maximum as the system can support only limited number of cells. This forces the population of cell vehicles to compete for resources. The vertical survival of the vehicle lineage depends on the competitive success of the vehicle. This indicates that for a replicator inhabiting the most successful vehicle *at the beginning of the competition* provides you with most descendants at the end of the experiment—unless, of course, the replicator can horizontally be transferred to other vehicles (as was argued above).

**Figure 3:**
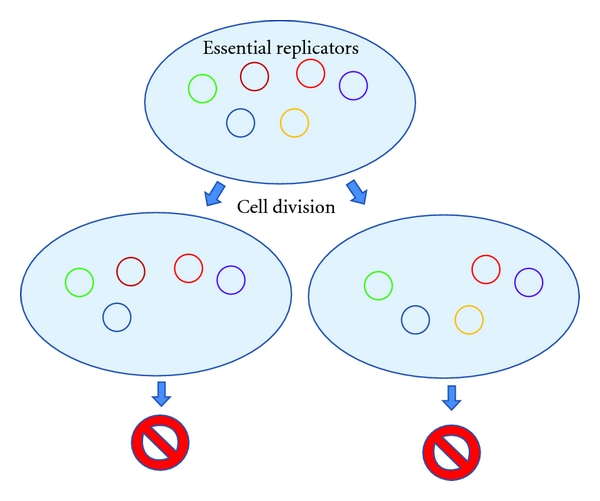
A cell vehicle, which contains its essential genetic information in multiple independent replicators, may be prone to lose some replicators during cell division and thus produce incompetent cells.

Now, for the sake of argument, let us play with this idea and consider a situation where all the genes within a cell vehicle are separate replicators (these being like very simple *Class II* plasmids). Each gene has a potential for being horizontally transferred between vehicles after cell destruction, but it also has a chance to become lost during cell-vehicle division (depicted in Figure 3). The reproductive success of the vehicle corresponds to the current combination of genes and other genetic information therein as they are responsible for the phenotype of the vehicle. Certain combinations are more successful than others, and therefore they have more descendants within certain timeframe. Some genes are essential for the survival and division of the vehicle, and thus loss of these replicators would terminate the vehicle lineage. Selection should focus on ensuring that the most essential genes are vertically stably maintained as any resources spent on an attempt to divide are wasted unless the essential genes are present in the new cell vehicle. Yet, maintenance of the faithful distribution of thousand individual molecules during a single cell division appears difficult to evolve or heavily costly (given that each of these molecules should have, e.g., an individual type of a segregation system or have regions for chromosome-like segregation), and selection should therefore intuitively progress towards the fusion of these genetic replicators into a single or as few molecules as possible (since this should help the robustness of the segregation during cell division). These replicators would be *Class I* replicators in the presented classification. This is very superficial analysis, yet, it might help grasp the idea that certain genetic functions need to be present within all vehicles at all times, and therefore they would be vertically inherited to all functional cell vehicles during vehicle division.

## 8. Replicators Evolving from One Class to Another

In Section 5 I briefly described few examples of replicators evolving into replicators of different *classes*. Now I will go through some examples where the ecological context favors the replicator to adopt the life strategy of replicators belonging to another *class*. Moreover, I will argue that the subsequent evolution of the replicator starts to favor phenotypes that resemble other replicators within its new *class*.

The general point for discussing this evolution is to illustrate that the classification can provide a framework for approaching complex evolutionary settings. Scientific classifications, however, may be harmful for profound understanding of systems, if we are unable to see beyond the classes themselves. Yet, I believe that a proper classification can give a simplifying touch on some of the acting forces of nature. It must be noted that the different classes of the presented classification do not have strict boundaries and replicators can readily change their classes. Still, the possibility to situate the replicators into these *classes* may reflect general evolutionary tendencies of complex microbial systems and thus prove practical in understanding microbial world.

### 8.1. Temperate Viruses Evolve into Virulent Viruses

Many bacteriophages are known to acquire mutations, which makes them unable to repress their lytic pathway [9, 24, 41]. These *Class IV* replicators lose their potential to exist vertically within a lineage of cell vehicles, and thus they transform into *Class V* replicators in the classification. These virulent mutants (or so-called clear plaque mutants) enter bacterial cells, replicate their genomes, express their structural proteins, assemble new virions, and lyse the cell. This evolution of *Class IV* replicators into *Class V* replicators is commonly used in bacteriophage research as the “new” *Class V* replicators are devoid of vertical survival within lineages, and therefore their fitness correlates only with their potential for replicating in other vehicle lineages. This, in turn, often increases the production rate of virions [24], which therefore helps conducting experiments that require virus particles. In other words and from the viewpoint of the classification, *Class IV *replicators started to approach the typical phenotypes of *Class V *replicators due to their incapability for vertical existence within a vehicle lineage.

### 8.2. Conjugative Plasmids Evolve into Nonconjugative Plasmids

Dahlberg and Chao, 2003, cultivated bacterial cell vehicles containing certain conjugative plasmids for 1100 generations (about half a year) [53]. The system did not contain plasmid-free vehicles, and therefore there was essentially no selection for maintaining the horizontal transfer potential of the conjugative plasmid. Indeed, it was observed that some of the *Class III* replicators had lost their potential for conjugation or the rate of conjugation had decreased during the 1100 generations of their host vehicles. Moreover, the reproductive cost of the plasmid had decreased significantly, indicating that selection efficiently focused on improving the vertical survival of the element within its current vehicle lineage.

After invading the whole population of cell vehicles, horizontal movement had no benefits for *Class III* replicator whereas the vertical survival improved its reproductive success. Therefore, the phenotype of *Class III* replicator in this study started approaching that of *Class II* and *Class I *replicators.

### 8.3. Temperate Viruses Evolve into Chromosomal Elements

Defective bacteriophages are abundant in many bacterial chromosomes [23]. What good does permanent colonization of a certain vertical lineage of cell vehicles do for *Class IV* replicator? Why not maintain the potential for forming the extracellular viral particle and thus the horizontal transfer potential? Indeed, it has been shown that bacterial genomes harboring functional prophages can have advantage over relatives that lack the phage [54].

Given the modern genomics, natural selection operating repeatedly on microbial communities appears to sometimes favor bacterial chromosomes that have defective bacteriophages integrated into them [23]. Naturally, there must be some reason why it is more favorable for the chromosome to maintain a defective provirus rather than a functional one. One possible (and obvious) explanation considers the differences between functional and defective proviruses. A functional provirus can occasionally induce its lytic activity and thus destroy the host cell vehicle (and the chromosome). Those cells that maintain a prophage are immune to infections by other similar viruses as these defective viruses can encode mechanisms that prevent superinfection, that is, multiple infections, of a single cell. However, given that the key elements for producing virions become in some way dysfunctional, then the defective virus becomes unable to destroy the host cell vehicle. In a population of cell vehicles where all chromosomes host a same provirus, then the ones hosting a defective provirus may have an advantage over the others [54].

Moreover, defective proviruses appear to start evolve a strictly vertical life strategy. Studies have demonstrated that the cost for carrying a provirus abates the longer the cells are grown in presence of the virus. Some of the proviral genes belonging to defective proviruses are still expressed within cells, suggesting that the provirus phenotype is benefitting only its present cell vehicle [23, 55]. This illustrates how replicators change their classes and utilize its previous genetic information in support to its new life style.

## 9. Why Antibiotic Resistance Genes Are Often Associated with *Class II* and *III* Replicators?

Why do bacteria help other, sometimes very distantly related, bacteria in their environment by sharing their antibiotic resistance genes with them? If you think that bacteria are generally competing with other bacteria for available resources, then it appears controversial to realize that the same bacteria are helping their rivals against antibiotic-producing organisms. Should it not be evolutionarily favorable for bacteria to let other bacteria die to antibiotics and thus allow them become the sole survivors of the system? This, however, is not the case when we observe bacteria in environments that are abundant with antibiotics. Have the bacteria allied against us just for the heck of it?

In order to realize why bacteria appear to be cooperating against our attempts to utilize antibiotics as an antimicrobial therapy, we must note that antibiotic resistance genes are often part of independent replicators which are not dependent on any particular bacterial cell [13, 16, 20, 21]. This scenario illustrates how and why the presented dissection of bacterial cell can be useful in comprehending bacterial evolution in environments where their evolution might be the matter of life and death.

It is known that majority of antibiotic resistance genes among clinical isolates of bacteria are actually part of conjugative or nonconjugative plasmids or transposons rather than being an inherent feature of any particular chromosome [16]. The spread of plasmids is considered the most common mean by which bacterial strains transform into drug-resistant phenotypes not only in clinical environments but also within other natural environments [20, 21, 38, 56, 57]. Indeed, antibiotic resistance provides a good example of natural selection where certain genes may become a part of horizontally moving replicators rather than vertical ones.

**Figure 4:**
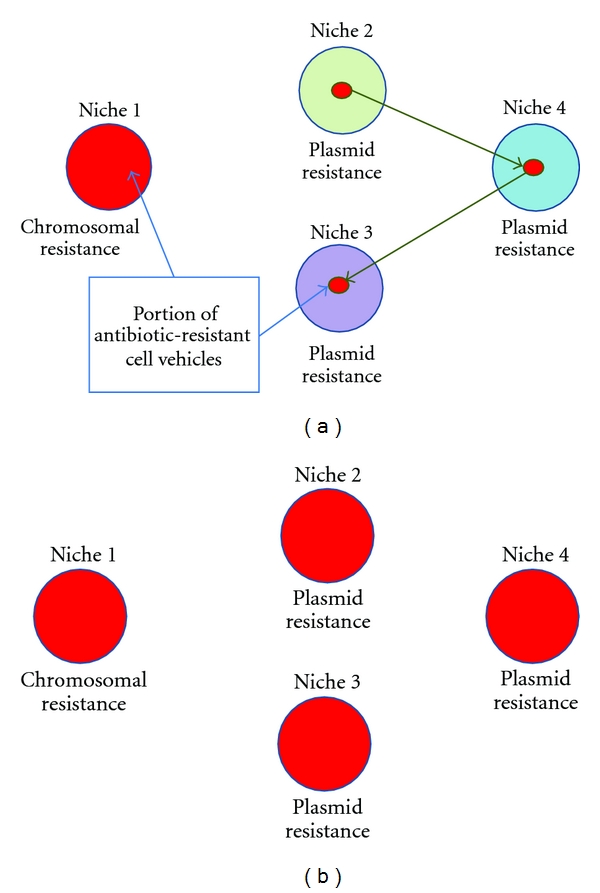
When plasmid- and chromosome-borne antibiotic resistances are compared, the plasmid-borne resistance can become more abundant after exposure to antibiotics.

Once again, I will present a hypothetical scenario (adapted from [58]) that may illuminate how natural selection results in rapidly spreading antibiotic-resistance genes within communities of competing bacteria (depicted in Figure 4). Imagine a system containing ten different bacterial species occupying their individual niches. Each of the bacterial lineages is well adapted to their own niche, and none of the other nine lineages are able to invade these niches. One of the nine vehicle lineages contains an antibiotic-resistance gene in its chromosome whereas one of the lineages contains a conjugative plasmid which carries the same antibiotic resistance gene. The conjugative plasmid poses a reproductive cost to its host cell vehicle, but it moves seldom to other lineages. The plasmid does not become prevalent in any single lineage due to the cost, but a portion of cell-vehicles in each of the lineages ends up harboring the plasmid at all times.

Now, an antibiotics-producing organism enters the environment and subjects all bacterial cell vehicles in all of the ten ecological niches to antibiotic selection. The bacteria will either die or suffer a significant reproductive cost due to the antibiotics that disrupt or terminate the functionality of the cell vehicles. Only those vehicles that happen to contain the antibiotic-resistance gene go unaffected by the antibiotics. The selection results in the death of majority of cell vehicles in the system, leaving room for the remaining cells to repopulate each niche.

Which cell vehicles are likely to occupy the free niches? In this scenario we can imagine two possibilities: either it is the cell vehicle that contains the chromosome with the antibiotic resistance gene or it is one of the cell vehicles that harbor the conjugative plasmid. The fitness of the cell vehicle in any of the niches is likely to correlate with its evolutionary history. In other words, cell-vehicles that previously occupied a certain niche are supposedly best adapted to that niche despite of the presence the plasmid in those vehicles. For this reason the vehicle population containing the chromosomal resistance gene might be unable to conquer any of the niches that suffered from the antibiotic selection despite the fact that the chromosomal resistance lineage itself was not affected by the selection. The result would be that nine of the ten niches became occupied by cell vehicles in which the conjugative plasmid is prevalent due to the opportunistic antibiotic resistance gene, and only one of the ten niches contained the resistance gene in the chromosome.

Horizontally spreading replicators, like plasmids and conjugative plasmids, might not be able to become abundant in cell-vehicle lineages due to their cost to the vehicle reproduction. They can, however, be present in multiple lineages as a minority. This minority of plasmid harboring vehicles with opportunistic genes can provide sudden boost to the vehicle fitness (as described above) and therefore become dominant in the population [58].

## 10. Do the Replicator and Vehicle Dependencies on Each Other Reflect General Evolutionary Tendencies?

**Figure 5:**
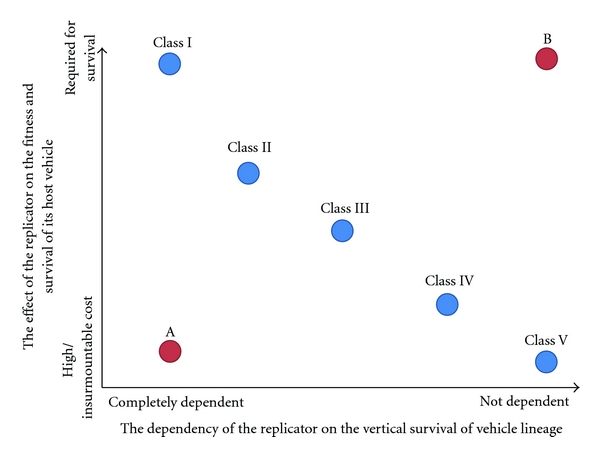
Positioning of the different *classes* of the classification into a two-dimensional plot where on one axis there is the horizontal movement potential of the class and on the other there is the effect of the replicator on its present cell vehicle.

As was argued in Section 4, replicators depend on the vertical survival of vehicles to various degrees. Similarly vehicles fail to survive in absence of certain replicators whereas they fail to survive in presence of other replicators. Chromosomes, for example, are fully dependent on their present lineages while virulent viruses are independent from any particular lineage of vehicles. This allows us to plot these dependencies on an approximate scale where on one axis there is the dependency of the replicators on vehicle lineages and on the other axis there is the effect of the replicator on the survival of its present vehicle (Figure 5). I will attempt to demonstrate that this plot may be useful visualization for approaching the evolution of prokaryotic replicators.

First, we observe that the more dependent a replicator is on a certain vehicle lineage, the more dependent a vehicle lineage is on the replicator. Second, we see that the more harmful a replicator is to a lineage, the less it depends on the survival of any particular vehicle. This correlation may appear to be a trivial tautology, but I suggest that, when we know the replicators' position on one axis, we also know its position on the other. I intend to state here that natural selection may be “aware” of this plot and therefore replicators generally evolve towards the corresponding position on the two-dimensional chart. In other words, if a vehicle cannot survive without some particular replicator, then selection favors changes that make it evolve towards a less horizontal form. Similarly, if a replicator is very costly in terms of reproductive success to its vehicle, then it survives best by being able to move horizontally between vehicle lineages or by evolving a less costly phenotype. While I will not attempt to prove this, I propose this as a hypothesis that may be used as a framework for predicting results of simulations or experiments and also for providing a general perspective on the evolution of prokaryotic biosphere.

For the sake of argument, I put letters A and B (representing imaginary replicators) on the plot (Figure 5) at positions that are free of *natural* replicators. Would it be possible that A and B actually existed in nature? I argue that the answer is negative. However, I want to emphasize that such replicators may, of course, exist transiently, but natural selection favors the change towards their correct positions on either of the axes or, alternatively, they will go extinct altogether. Therefore replicators A and B are not evolutionarily stable replicators with their present life strategies.

Replicator A decreases the reproductive fitness of its vehicle. Therefore any vehicle in the environment that lacks A is able to outreproduce vehicles containing A, leading into the extinction of A. However, A could also achieve potential to be transferred horizontally between vehicle lineages (by recombining with a conjugative plasmid, e.g.), which would make A less dependent on the survival of its current lineage. This means that A would be likely to move rightwards on the two-dimensional plot. The other possibility is that A could evolve into a less costly replicator, making it move upwards on the plot. You may consider a host-destroying virus that makes defective virions as an example of A. This virus should evolve either a phenotype that does not destroy the vehicle or it should form functional virions in order to survive.

The case of replicator B is a somewhat less obvious one. B is essential for its present vehicle, but it is not dependent on the vertical survival of any particular vehicle lineages. In other words, vehicles require B for survival, but B itself can freely move between vehicles. However, if we think of the situation, we realize that (by definition) all vehicles must contain a copy of B in order to survive and reproduce. Therefore B would be present in every single surviving vehicle lineage, and the horizontal movement potential would pose only an unnecessary reproductive cost for the current vehicle. From this perspective it appears logical that B will lose its horizontal movement potential as selection would favor the nonhorizontal and therefore less costly phenotype.

A and B depict two unnatural cases, but they provide an example how natural selection may be operating against these positions in the plot. However, the situation becomes increasingly more difficult when we consider any intermediates between A and B. In natural environments and ecological communities, the position of the replicator on *y*-axis is likely to constantly change depending on the current surrounding conditions of its present vehicle. In presence of antibiotic-producing organisms, the plasmid providing the resistance might be essential for the cell, but this essentiality ceases when the antibiotic producing organism disappears from the effective area of the vehicle. What is the position of such a replicator on *y*-axis? Similarly certain replicators might be relatively costly to their host vehicles, but they can sometimes give huge advantage to their vehicles due to seldom occurring conditions. However, the diversity and the complexity of natural environments is vast, and it is easy to get lost into the ocean of details. For this reason the plot should be seen as a tool, which may allow approaching complex phenotypes of multiple different types of replicators from a very general perspective.

## 11. An Example Case of the Emergence of a Relevant Bacterial Organism through Accumulation of Multiple Replicators into a Single Vehicle

Vehicle concept and replicators can provide a general way to approach and explain changing behaviors of bacterial organisms. Microbial world is often seen to consist of just bacteria (and archaea) and viruses. These microbes are living on this planet in any suitable habitat, that is being anything from a rectum of an animal to a hydrothermal vent in the mid-Atlantic ridge. This view is not wrong, and indeed it is the one that we observe with our microscopes. Similarly, general books about microbes generally describe a variety of different viruses and prokaryotes with their taxonomic families and evolutionary relationships. However, these books often credit other horizontally moving replicators to lesser extent despite the fact that they may play a significant role in biological systems and that they are arguably distinct entities in respect to any particular bacterium. Moreover, the general view fails to distinguish the different roles of temperate and lytic viruses. Indeed, the chimerical reality of multiple intercellular and extracellular replicators is a fundamental part of bacterial life, and thus acknowledging this diversity can help us realize why and how certain microbial organisms arise.

What kind of an organism was the enterohemorrhagic *Escherichia coli* (EHEC) that was responsible for the outbreak in Germany in 2011 and that tragically killed tens and caused a severe disease in thousands? From the perspective of this paper, it is interesting that replicators of various classes of the presented classification played a role in the outbreak [59].

Mainstream media described EHEC as a common human bacterium that happened to cause a dreadful disease. To people who are unaware of the details of microbial world, the overall image must have been that *Escherichia coli* can sometimes become extremely harmful. How do some of these naturally commensal bacteria happen to turn into hazardous or even lethal pathogens? Naturally, the evolution of virulence is a complicated matter with a variety of affecting factors. Yet, for a realistic approach, we must understand that it can be independent genetic elements that are responsible for forcing the commensal bacterial organisms to turn into the causative agents of epidemics. Indeed, sometimes news articles about EHEC mentioned that bacteria naturally swap genes with each other and this exchange is behind the emergence of this new lethal version of the bacterium. However, it may still appear unclear how bacteria know to transfer these nasty genes into other bacteria and why do they do that. As in case of antibiotic resistances, for profound understanding we must realize that it is not any actual bacterium transferring these genes, but instead that a bacterium is an organism that consists of a cell vehicle along with a chromosome and possibly some other genetic replicators. And these other replicators are the ones that induce the phenotype that is responsible for transferring horizontally genes into other bacteria. And they do it because it is beneficial for their own survival and reproduction.

EHEC behind the Germany outbreak contained temperate viruses (*Class IV*) that provided the Shiga toxin genes responsible for the pathogenic phenotype of the bacterium [60]. In other words, EHEC would not have caused the epidemic if there were no *Class IV* replicator that used the same cell vehicle with the chromosome for its propagation and preservation. Moreover, EHEC strain contained a large conjugative plasmid (*Class III*) that provided the vehicle with antibiotic resistances and some other useful phenotypes. However, EHEC infections are usually not treated with antibiotics anyway as antibiotics may increase Shiga toxin production of the bacterium. Nevertheless, the plasmid may have given the vehicle a potential to survive in environments where it would have naturally succumbed. Overall, by realizing that bacterial cells are combinations of various independent genetic entities, we may understand how new diseases and super bugs emerge from previously harmless organisms.

## 12. Examples of Using the Classification in Formulating Hypotheses for Evolutionary Experiments

I want to demonstrate that the presented classification could be used to provide a framework for formulating some practical scientific hypotheses (e.g., predicting outcomes of evolution experiments). I give few simple examples that essentially ask whether or not we may approach evolving bacterial populations from the viewpoint of various replicators with differing potentials for horizontal movement and with differing effects on the survival of the cell vehicles.

*Opportunistic genes that only sometimes but significantly improve the survival or reproductive rate of a cell vehicle are likely to become associated with horizontally moving replicators rather than Class I vertical replicators in natural communities of bacteria.* In principle, this hypothesis could be tested by cultivating a diverse bacterial population in an environment where there are multiple niches available. One of the bacteria would contain the opportunistic gene (like antibiotic resistance) in its chromosome replicator, and one of them would have the gene in a horizontally moving replicator (like conjugative plasmid). The system would be let to grow for some time before and after introducing the antibiotic selection to the system. The prevalence of the opportunistic gene in horizontal replicator instead of the chromosomal replicator could be measured.

*If an opportunistic gene associated with a horizontally moving replicator becomes mandatory for the survival of the cell vehicles in the environment, then the replicator associated with the opportunistic gene evolves towards a (more) vertical phenotype or the gene becomes part of one of the vertical replicators.* In principle, the hypothesis could be tested by introducing a conjugative plasmid (containing an opportunistic gene, like antibiotic resistance) to a population of bacteria. Then, lethal doses of antibiotic selection would be stably maintained in the system over several bacterial generations. After the selection, the cost of the plasmid to the cell-vehicle and its conjugation rate could be measured.

*If selection focuses against a replicator on which the cell vehicle is not dependent, then the complete replicator can become eliminated. If selection focuses on an essential replicator, then the replicator is likely to only change its phenotype or the whole vehicle lineage becomes terminated.* This was the actual hypothesis in a recently published experimental paper by me and my colleagues [13]. We tested what happens when plasmid-dependent phages were cultivated with bacteria harboring plasmids in presence and absence of the selection for the plasmid. In absence of the selection, the plasmid was shown to become lost. In presence of the selection, the plasmid (or the selected parts of it) survived but its phenotype changed.

## 13. Host Range and the Replicators in the Evolution of Biospheres

In this final section I will consider what the possibility to classify replicators according to their effects on the survival of host cell vehicle and their horizontal movement potential might implicate about the evolution of vehicle- and replicator-based biospheres. For those interested in pondering the development of hypothetical forms of life, this discussion can serve as a (testable) hypothesis about the general trends in the evolution of any living system in this universe.

Replicators that move between vehicles have varying host ranges. By the term host range is meant the portion of cell vehicles into which a replicator can transfer to and subsequently replicate in. A virulent virus can usually infect only a tiny fraction of closely related cells whereas conjugative plasmid can be transferred successfully to a much wider range of unrelated cells. The host range of virulent viruses is narrow whereas the host range of a plasmid is large. Naturally, this is not a coincidence.

Virulent viruses terminate the cell vehicles wherein they replicate. Therefore all the other replicators, especially chromosomes, become eliminated due to virus replication. Selection therefore favors those chromosomes among a population of cell vehicles that produce phenotypes which are unrecognizable by viruses. This has been confirmed in various studies that demonstrate the coevolutionary arms race between viruses and their hosts [61]. On the other hand, conjugative plasmids have been shown to be able to transfer and replicate in a variety of different types of cells. There is stronger selection pressure for chromosomal replicators to avoid viruses than to avoid plasmids. Sometimes avoidance of a plasmid can be lethal whereas avoidance of a virus is rarely harmful. In other words, evolutionary dynamics, in general, force the replicators with higher cost on the host cell vehicle to have narrower host range. Now, it can be asked whether this notion may provide any insights about evolving biosystems. There are already numerous papers about coevolutionary dynamics of viruses and cells [61], about virus-driven evolution [62], and about host ranges [63]. My intent is not to repeat them but instead to try applying a more general perspective on the issue.

Our biosphere is abundant with all the types of replicators of the proposed classification, and therefore we may not consider it relevant to think whether or not this is mere coincidence or a direction towards which *any given biosphere* progresses. But what if we take another independently emerged and evolved (although hypothetical) living system which contains vehicles and replicators? If we go through the replicators in that system, are we able to use this same classification for them as we are for replicators on Earth? Do all the *classes* have at least some representatives in the foreign biosphere? Or are there systems where, for example, only chromosomes or just chromosomes and plasmids thrive?

We need to note that the considered biosphere must be large enough in order for this question to be relevant. When we take a small sample of microbes in our world, we may find that some of the replicators, like conjugative plasmids, cannot be found. Therefore tiny cellular communities may not be able to support the full variety of replicators. But what is the case when we take, let us say, a planet full of microbial life? Can we say with relative certainty that we are going to find plasmids, conjugative elements, and viruses just because that is how natural selection in general tends to shape evolving biospheres that are abundant with single-celled organisms?

In order to approach this question, we may consider biospheres where replicators of some of the classes are absent and evaluate whether or not it is possible that some other replicators will inevitably evolve to represent the missing *class*. In Section 10, I argued that replicators may be evolving towards the *correct *position on the two-dimensional plot presented in Figure 5. Now, if one of the classes depicted in Figure 5 had no representatives in a given biosphere, like there were no *Class V* replicators at all, would some of the other replicators be likely to evolve to fill this free *niche*? I will not go through all the possible cases or scenarios but instead address few general ideas.

If a foreign biosphere completely lacked viruses (that can directly cause the demise of their hosting cell vehicles), what would likely to be different in comparison to our biosphere? Naturally, one can think of a huge number of things. However, perhaps one of the most relevant for our considerations is the notion that there would be no evolutionary arms race between viruses and hosts. Cellular populations would not need to maintain variation against constantly evolving virosphere, and, therefore, in absence of viral-induced selection for variance there might be a huge number of cells that maintain, for example, highly conserved surface components. This could indicate that if a virus emerged, it would be likely to be able to reproduce within a huge population of hosts. In other words, any crudest form of a virus would be likely to have a very wide host range and thus be highly successful in producing copies of itself. Therefore, the naivety of the biosphere due to the lack of previous exposure to viruses might render it highly vulnerable to viral invasion. Given a large biosphere and long-enough timeframe, viral strategy might be bound to emerge sooner or later. Experiments have shown that bacterial populations unexposed to viral selection tend to be more homogenous in comparison to those with viral predators [62].

What if a system had viruses and chromosomes but was devoid of plasmids? Would plasmids be likely to emerge? To address this question, we may need to consider what the usual characteristics of plasmids in our biosphere are, and then ask whether these characteristics should also become associated with plasmid-like replicators (with higher horizontal movement potential than chromosomes) in any other biosphere. Indeed, plasmids often appear to harbor opportunistic genes, like those conferring antibiotic resistance. Such genes may also be likely to exist in foreign living systems, given that biospheres anywhere should inhabit environments where selection pressures are likely to change according to the current ecological and environmental conditions of the particular cell vehicles. If such opportunistic genes are present, then by reconsidering the mind exercise presented in Section 6 and Figure 4, we may find it logical that *Class II* or *Class III* like replicators may emerge due to local evolutionary dynamics. In other words, opportunistic genes may provide an evolutionarily favorable path for the appearance of smaller low-cost replicators that have increased potential for horizontal movement.

In more general terms, I suggest that it is possible that in large biospheres evolution may progress towards various types of replicators with varying potential for horizontal movement, perhaps even to fill all the slots in the presented classification. Naturally, this suggestion can and must be subjected to variety of different types of experimental tests. Nevertheless, in our biosphere all the different classes appear to be evolutionarily stable strategies as they are abundant and ancient. Therefore, given a sizable enough frame from which to observe evolving systems with cell vehicles and replicators, similar stability may be inevitable to emerge. However, it is still very much possible that these classes may be a feature solely of our type of microbial life. Either way, improved knowledge of the underlying issues would help us understand evolving systems nonetheless.

Finally, I want to emphasize that all of the replicator types we now observe in our biosphere may have emerged before the formation of the first consistently reproducing cell vehicle and chromosome. However, discussing the emergence of all the *classes* as a part of an evolving primordial community is far beyond the scope and length of this paper (although being previously discussed to some extent [14, 15, 64–66]). It may, nevertheless, be possible that the early evolutionary dynamics of emerging life anywhere in this universe may naturally generate replicators with varying potential for horizontal movement between cell vehicles. And as the life advances, the replicators remain as a permanent part of the system.

To conclude, horizontal movement and replicator phenotypes may be approached from a general perspective where we do not pay attention to exact details but rather observe the overall characteristics of replicators in an attempt to understand why and how evolving systems, such as prokaryotic biospheres, may appear to be constructed the way they are. At this time, however, it might be impossible to say whether or not this would be of any practical use or lead to meaningful insights.

## Figures and Tables

**Table 1:** Classification of replicators.

Class	Example replicators	Vertical dependency	Horizontal movement potential	Description of average phenotypes
I	Prokaryotic chromosomes	*Completely dependent *	*No potential*	Encodes the main functional units of all cell vehicles. Required for the binary fission of the cell vehicle.

II	Plasmids, transposons	*Highly dependent*	*Passive *	Low reproductive cost to host cell vehicle. Can encode opportunistically useful phenotypic traits.

III	Conjugative plasmids, integrative and conjugative elements (ICEs)	*Moderately dependent* (always requires a cell vehicle)	*Active* without an extracellular stage	Moderate or low reproductive cost to host cell vehicle. Usually encode opportunistically useful phenotypic traits.

IV	Temperate viruses	*Somewhat dependent* (can survive even if the cell-vehicle terminates)	*Active* with an extracellular stage	Moderate or low reproductive cost to host cell vehicle. Sometimes encode opportunistically useful phenotypic traits.

V	Virulent viruses	*Not dependent *	*Active* with an extracellular stage	Insurmountable reproductive cost that terminates the host cell vehicle. Does not encode cell-vehicle benefitting traits.
